# METTL16 in cancer: Roles and regulatory mechanisms

**DOI:** 10.1016/j.gendis.2025.101758

**Published:** 2025-07-04

**Authors:** Kangjie Qiu, Shuxin Zhong, Jinyi Liu, Weini Li, Cunte Chen, Yangqiu Li, Chengwu Zeng

**Affiliations:** a Key Laboratory for Regenerative Medicine of Ministry of Education, Institute of Hematology, School of Medicine, Jinan University, Guangzhou, Guangdong 510632, China; b Guangzhou First People's Hospital, The Fifth Affiliated Hospital, Guangzhou Medical University, Guangzhou, Guangdong 510700, China; c Cedars-Sinai Cancer Institute, Department of Biomedical Science, Cedars-Sinai Medical Center, Los Angeles, CA 90048, USA; d Jinan-Puhua Joint Laboratory, Guangzhou, Guangdong, China

**Keywords:** Cancer, m^6^A, METTL16, RNA methylation, SAM metabolism

## Abstract

The regulation of gene expression is pivotal in cancer development, with increasing emphasis on epigenetic modifications such as RNA methylation. N6-methyladenosine (m^6^A), the most abundant RNA modification, critically impacts RNA function and stability. METTL16, an m^6^A methyltransferase or “writer”, is essential in this modification process. Aberrant expression of METTL16 is closely linked to cancer cell proliferation, invasion, metastasis, and drug resistance, through modulation of RNA metabolism. Despite extensive research on RNA-modifying enzymes, the specific mechanisms and roles of METTL16 in cancer remain poorly understood. This review provides a detailed examination of METTL16's functions and regulatory mechanisms in cancer, emphasizing its m^6^A-dependent and m^6^A-independent roles in regulating RNA stability and function. Furthermore, it proposes that targeting METTL16 represents a promising avenue for cancer therapy.

## Introduction

The regulation of gene expression plays a critical role in cancer development, drawing increasing attention in recent years.^1^ The onset and progression of cancer are not only associated with genetic mutations but also involve complex gene expression regulatory mechanisms, particularly through epigenetic modifications. Epigenetic modifications influence gene activity and expression without altering the genetic sequence, primarily through chemical modifications, among which RNA methylation is pivotal.^2^ These modifications include N6-methyladenosine (m^6^A), N1-methyladenosine, 5-methylcytosine, and N7-methylguanosine, with m^6^A being the most abundant and critical RNA modification in mammals.^3^^,^^4^

The m^6^A modification is recognized as a reversible methylation process regulated by an ensemble of methyltransferases (“writers”), demethylases (“erasers”), and methyl-binding proteins (“readers”), collectively known as m^6^A regulatory factors. The writers, such as methyltransferase 3 (METTL3) and METTL14, are responsible for catalyzing the formation of m^6^A, significantly impacting RNA function and stability. METTL3 and METTL14 have been extensively studied for their roles in this modification process, establishing a foundational understanding of m^6^A dynamics.^5^ In contrast, other writers, like METTL16 and zinc finger CCHC-type containing 4 (ZCCHC4), exhibit a preference for binding to RNA with specific sequence and structural features, whereas METTL3/14 show less dependence on such structural elements.^6^^,^^7^ Among the m^6^A writers, METTL16 is particularly noteworthy for its crucial role in RNA modification and transcriptome regulation. Like METTL3, METTL16 is active in both the nucleus and cytoplasm, where it participates in RNA biosynthesis, degradation, and translational regulation. METTL16 influences various stages of gene expression through its m^6^A modification activity.^8^, ^9^, ^10^ Aberrant expression of METTL16 has been closely linked to tumor proliferation, invasion, metastasis, and resistance to chemotherapy.^11^, ^12^, ^13^, ^14^, ^15^ Depending on the context, METTL16 can either promote or inhibit tumorigenesis by regulating the expression of key oncogenes and tumor suppressor genes. Additionally, METTL16 can modulate tumor progression by altering the structural and functional integrity of the genome, further emphasizing its potential as a pivotal regulator in cancer biology.

Despite extensive research into various RNA-modifying enzymes in cancer, the specific roles and mechanisms of METTL16 remain inadequately understood. This article aims to comprehensively summarize the role and regulatory mechanisms of METTL16 in cancer, emphasizing its m^6^A-dependent and m^6^A-independent functions in the regulation of RNA function and stability. It suggests that targeting METTL16 could be a promising strategy for cancer prevention and treatment.

## The structure of METTL16

**Figure 1:**
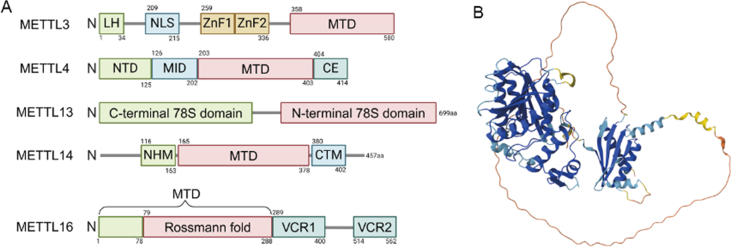
Structure of the METTL protein family. **(A)** The METTL protein family, comprising METTL3, METTL4, METTL13, METTL14, and METTL16, is characterized by distinct structural domains that confer specific functional roles. **(B)** Structure prediction of METTL16 from the AlphaFold project. LH, leading helix; NLS, nuclear localization signal; ZFD, zinc finger domain; MTD, methyltransferase domain; NTD, N-terminal domain; MID, the middle domain; CE, C-terminal extension; NHM, N-terminal α-helical motif; CTM, C-terminal motif; VCR, vertebrate-conserved region.

Full-length METTL16 consists of 562 amino acids and is characterized by two main domains: the N-terminal core methyltransferase domain (MTD) and the C-terminal vertebrate-conserved regions (VCR1 and VCR2) (Fig. 1A, B). Initial studies combining size-exclusion chromatography with small-angle X-ray scattering suggested that full-length human METTL16 exists as a dimer.^16^ However, subsequent experiments using size-exclusion chromatography coupled with multi-angle light scattering revealed that the wild-type, full-length METTL16 appears to exist as a monomer in solution.^6^^,^^17^^,^^18^ The measurements of multi-angle light scattering, which are independent of biomolecular shape and provide absolute molecular mass, are considered more reliable than small-angle X-ray scattering for determining oligomeric states.^8^^,^^19^ These contrasting findings highlight the complexity of METTL16's oligomeric state, which warrants further investigation to resolve the discrepancy between the techniques.

The MTD contains a characteristic Rossmann fold spanning residues 79 to 288, which is essential for binding S-adenosylmethionine (SAM) and RNA. This fold is composed of seven tightly packed β-strands alternating with α-helices. The β-strands form the structural core, providing a framework for the SAM-binding pocket, while the alternating α-helices intercalate between the β-strands to create a cavity for SAM and RNA binding.^6^^,^^16^^,^^20^ These structures are stabilized by hydrogen bonds and hydrophobic interactions, ensuring precise positioning of SAM and RNA within the catalytic pocket.^21^^,^^22^ Key residues within the Rossmann fold, such as Asn184, Pro185, and Pro186, contribute to SAM binding and stabilization through hydrogen bonds and hydrophobic interactions.^16^^,^^23^ Upon SAM binding, the Rossmann fold undergoes subtle conformational adjustments to enhance binding stability. RNA binding, on the other hand, is facilitated by positively charged residues on the fold's surface, including Arg82, Lys251, and Lys252, which use electrostatic attraction and hydrogen bonding to correctly position RNA. These structural features allow METTL16 to specifically recognize RNA substrates, such as methionine adenosyltransferase 2A (MAT2A) mRNA. Compared with the METTL3/METTL14 complex, METTL16 relies on its unique Rossmann fold to identify distinct RNA substrates.^22^ MTD is the primary site for methyl transfer activity, capable of binding to SAM and catalyzing the transfer of a methyl group from SAM to RNA, resulting in m^6^A modification. The asparagine-proline-proline-phenylalanine (NPPF) motif is a highly conserved and functionally critical component of the MTD. This conserved amino acid sequence is located at the catalytic site responsible for binding to SAM. The NPPF motif likely facilitates SAM recognition and binding by shaping the spatial configuration of the catalytic site. When intact, the NPPF motif ensures high catalytic efficiency during SAM binding. However, mutations in this motif, such as substituting NPPF (Asn184, Pro185, Pro186, and Phe187) with 2-acetamido-3-phenylpropanoic acid (APPA), significantly impair SAM binding and reduce the efficiency of substrate methylation.^6^^,^^24^ The K-loop is a self-regulating peptide loop in METTL16's structure that orderly closes in the presence of RNA, shielding the SAM binding site. This closed state is stabilized through hydrophobic interactions with the core MTD, with the critical lysine residue (Lys163) occupying the cofactor binding pocket, thus diminishing METTL16's activity. In the absence of RNA, the K-loop adopts a different conformation, where Lys163 is exposed to the solvent and other hydrophobic side chains, such as Met167, are distant from the domain, indicating that the closure of the K-loop is closely related to RNA binding status.^6^ Preceding the Rossmann fold is a unique region defined by N-terminal residues 1–78, which includes three α-helices and two short β-strands. This region contains positively charged residues such as Lys5, Lys14, Arg10, Arg12, Arg41, Arg47, and Arg82.^16^^,^^18^ These residues form a groove that effectively anchors the target RNA, creating the spatial conditions necessary for methyl transfer and playing a crucial role in interactions with specific RNA substrates.^18^

VCR1 and VCR2 are separated by predicted disordered regions, playing critical roles in various biological processes, including RNA methylation and intracellular signal regulation.^11^^,^^16^ They fold into β-sheets containing five β-strands and are similar to the kinase-associated-1 (KA1) domain of the specific U6 small nuclear RNA (snRNA)-terminal uridylyl transferase 1 (RNU6-1).^25^ This structural feature allows METTL16 to maintain complex RNA interactions in higher vertebrates. Over 75% of METTL16 peak observations occur in intronic regions, a proportion significantly higher than that shown for METTL3. Current evidence indicates that METTL16 mainly influences 5′ splice-site selection in the spliceosome by depositing m^6^A at the A43 position of U6 snRNA; it does not itself directly participate in the splicing processing of U6 snRNA.^10^^,^^22^^,^^26^

The UACAGARAA (R: G or A) consensus sequence is a key RNA motif recognized and methylated by METTL16, with the target adenine (A) typically methylated at a specific position, such as the second adenine in “UACm^6^AGARAA”. This sequence is prevalent in MAT2A mRNA and other potential METTL16 targets, often forming methylation sites in exposed regions of RNA secondary structures, such as stem-loops.^27^ In these structures, the UACAGARAA sequence is positioned in the exposed loop, enabling METTL16 to bind more easily and precisely target the adenine for methylation. The exposed adenine interacts with conserved motifs in METTL16, such as NPPF, through hydrogen bonding and stacking interactions. The transition zone between the stem and loop is critical for ensuring proper loop conformation. Conserved base pairs and specific hydrogen bond networks in this region enhance substrate affinity and methylation efficiency. Most identified METTL16-dependent m^6^A sites reside in introns, where they can influence pre-mRNA splicing, thereby affecting gene expression (see the subsection “m^6^A-dependent mechanisms in mRNA regulation” for a MAT2A-specific example).^11^^,^^22^ During methylation, the target adenosine must be unpaired and surrounded by stem-loop structures, where prominent nucleotides significantly affect methylation efficiency. Such information is commonly used to predict the methyltransferases required for specific m^6^A events based on sequence and structural context.^10^^,^^18^ The enzymatic activity of METTL16 strictly depends on specific target sequences and their secondary structural features, which is based on comprehensive *in vitro* and *in vivo* analyses of methylation sites in MAT2A and U6 RNA.^11^^,^^28^^,^^29^

## Comparative functional redundancy and specificity of METTL16 versus METTL3/14

Conducting a detailed comparison of METTL16 with the canonical METTL3–METTL14 complex is critically important for uncovering the distinctive functions that METTL16 exerts in tumor initiation and progression. Table 1 summarizes the biochemical properties, sub-cellular localization, and substrate preferences of the principal human m^6^A writers, providing the foundation for the discussion below. Although both m^6^A-dependent and m^6^A-independent functions of METTL16 have been reported, its functional overlap with the canonical METTL3/14 complex, as well as the distinct features that differentiate METTL16, remain incompletely understood.

**Table 1:** Summary of the characteristics of human m^6^A RNA methyltransferase.

m^6^A MTFase	Subcellular localization	Main regulatory RNA	Depends on the secondary structure	mRNA location for m^6^A	Catalytic sequence
METTL3	Nuclear speckles, nucleus, and cytoplasm in some cancers	mRNA, lncRNA, miRNA	Yes	Enriched near stop codons and in 3′ UTRs	DRACH
METTL14	Nuclear speckles, nucleus	None, catalytically inactive	No direct catalysis	Enriched near stop codons and in 3′ UTRs (due to METTL3 activity)	DRACH
METTL16	Nucleolus, nucleus, cytoplasm	U6 snRNA, MAT2A mRNA, lncRNA MALAT1	Yes	Varies, can target structured regions in untranslated regions and introns	ACAGAR in stem-loop or bulge

The METTL3/14 complex functions as a heterodimer to deposit m^6^A modifications on mRNAs and precursor miRNAs. In this complex, METTL3 provides the catalytic activity, METTL14 enhances RNA binding stability, and Wilms tumor suppressor-1-associated protein (WTAP), along with associated adaptor proteins, facilitates localization to nuclear speckles. The METTL3/14 complex preferentially recognizes the DRACH consensus motif and operates largely independently of RNA secondary structure.^21^^,^^30^^,^^31^ In contrast, METTL16 operates either as a monomer or a homodimer and does not require additional cofactors. Its N-terminal RNA-binding domain, in conjunction with a C-terminal arginine-rich vertebrate-conserved region, confers specificity for particular RNA structures, such as defined hairpins. This structural specificity results in a largely non-overlapping set of RNA substrates compared with the METTL3/14 complex. When both methyltransferases act on the same transcript, their modification sites are spatially distinct. Additionally, METTL16 contains an autoinhibitory K-loop that regulates the binding of SAM, a regulatory element absent in the METTL3/14 complex.^6^

Beyond methylation, the two systems diverge mechanistically. In gastric cancer, METTL3 interacts with poly(A) binding protein cytoplasmic 1 (PABPC1) and stabilizes its association with the cap-binding complex eukaryotic translation initiation factor 4F (eIF4F), whereas METTL16 directly interacts with the translation repressor eIF4E2 in lung cancer.^32^^,^^33^ Unlike the METTL3/14 complex, METTL16 displays distinct methyltransferase-independent activities, highlighting its broader functional versatility. Nuclear METTL16 additionally acts as a splicing enhancer for the final intron of MAT2A and limits DNA end resection after damage.^34^ Consistently, RNA interactome profiling has identified thousands of METTL16-bound transcripts, yet only a small subset displays m^6^A modification. To date, over ten direct targets have been validated using methods such as miCLIP and SCARLET, including MAT2A mRNA, U6 snRNA, branched chain amino acid transaminase 1/2 (BCAT1/2), suppressor of glucose autophagy-associated 1 (SOGA1), and ferredoxin 1 (FDX1) (Table 3). This highlights METTL16's dual role as both an m^6^A writer and a versatile RNA-binding regulator.^35^

Evidence from cancer models further emphasizes the non-redundancy between METTL16 and METTL3/14. In acute myeloid leukemia, the METTL3/14 complex promotes leukemogenesis through m^6^A deposition. However, acute myeloid leukemia cells are even more dependent on METTL16. Deletion of METTL16 suppresses proliferation, induces differentiation and apoptosis, and almost completely abolishes xenograft growth, while sparing normal hematopoietic stem cells. This contrasts with the broader hematopoietic defects caused by METTL3 loss.^36^, ^37^, ^38^ In hepatocellular carcinoma, METTL3 is frequently overexpressed and oncogenic, and METTL14 is often down-regulated and acts as a tumor suppressor.^39^^,^^40^ METTL16 displays a context-dependent mix of catalytic and non-catalytic functions. Under conditions of elevated intracellular iron, METTL16 exerts its methyltransferase activity by depositing m^6^A on the SUMO-specific peptidase 3 (SENP3) transcript, thereby stabilizing its expression and suppressing ferroptosis. In contrast, under conditions such as low SAM availability or hypoxia, METTL16 primarily functions through non-catalytic RNA-binding mechanisms.^41^^,^^42^ These findings indicate that METTL16 and the METTL3/14 complex exert distinct, context-specific effects, thereby positioning METTL16 as an attractive therapeutic target with a potentially favorable safety profile.

## METTL16 in cancer

### Expression patterns and prognostic significance

The expression patterns of METTL16 have been extensively studied across various cancer types, revealing its dual role in clinical outcomes. Depending on the cancer context, METTL16 expression can be either up-regulated or down-regulated, serving as a prognostic biomarker. In general, higher METTL16 expression is associated with poor clinical outcomes, including reduced overall survival, shorter disease-free survival, and increased metastasis risk. However, in certain cancer subtypes, elevated METTL16 expression correlates with more favorable clinical outcomes, highlighting its context-dependent role. This dual functionality is influenced by tumor type, genetic background, and interactions with other molecular pathways, underscoring the complexity of METTL16 in cancer progression.^43^

A recent study identified METTL16 as the most crucial METTL protein for cancer survival out of 25 METTLs.^26^ High METTL16 expression is associated with poor prognosis in several cancers, including gastric cancer,^43^, ^44^, ^45^ hepatocellular carcinoma,^26^^,^^46^^,^^47^ esophageal cancer,^48^ colorectal cancer,^49^, ^50^, ^51^ lung cancer,^32^ glioblastoma,^52^^,^^53^ melanoma,^54^ osteosarcoma,^55^ breast cancer,^56^^,^^57^ cholangiocarcinoma,^58^ and leukemia.^36^^,^^59^ Conversely, lower METTL16 expression is observed in pancreatic ductal adenocarcinoma,^34^^,^^60^ endometrial cancer,^61^^,^^62^ epithelial ovarian cancer,^63^ papillary thyroid carcinoma,^15^^,^^64^ and urothelial carcinoma,^65^ which are associated with poor prognosis. These divergent expression patterns emphasize the complexity of METTL16's role in cancer and its potential as a prognostic biomarker for patient stratification and treatment decisions. Table 2 presents a comprehensive summary of expression patterns, functional polarity, and representative mechanisms in each cancer type.

**Table 2:** METTL16 expression, functional polarity, and representative mechanisms across human cancers.

Cancer types	METTL16 level	Prognosis	Functional role	Representative mechanism	References
Gastric cancer	High	Poor overall survival; advanced stage and lymph node metastasis	Oncogenic	K229 lactylation boosts m^6^A on FDX1 → regulation of cuproptosis	43, 44, 45
Hepatocellular carcinoma	High	Poor overall survival and disease-free survival; higher tumor grade, vascular invasion, and early recurrence	Oncogenic	Cooperates with METTL3 to stabilize ZNNT1; eIF3-driven translation enhancement	26,46,47
Esophageal cancer	High	Poor overall survival; diminished response to immune checkpoint inhibitors	Oncogenic	High expression correlates with immune checkpoint resistance	48
Colorectal cancer	High	Poor overall survival; frequent metastasis and chemoresistance	Oncogenic	Stabilizes SOGA1, activates PDK4 → glycolytic shift	49, 50, 51
Lung cancer	High	Poor overall survival; contributes to drug resistance	Oncogenic	Sequesters eIF4E2, favors cap-dependent translation of oncogenic mRNAs	32
Glioblastoma	High	Poor overall survival; associated with isocitrate dehydrogenase (IDH)-wild-type and mesenchymal subtype	Oncogenic	Up-regulates NFE2L2 signaling, alters immune infiltration	52,53
Melanoma	High	Poor overall survival; correlates with advanced stage	Oncogenic	Mechanistic details pending	54
Osteosarcoma	High	Poor overall survival; linked to chemotherapy resistance	Oncogenic	Mechanistic details pending	55
Breast cancer	High	Poor overall survival and relapse-free survival; promotes metastasis	Oncogenic	Increases m^6^A and translation of GPX4, suppressing ferroptosis	56,57
Cholangiocarcinoma	High	Poor overall survival; correlates with gemcitabine resistance	Oncogenic	Mechanistic details pending	58
Leukemia	High	Poor overall survival; high relapse risk	Oncogenic	Maintains BCAT1/2-driven branched-chain amino acids metabolism and leukemia-stem-cell self-renewal	36,59
Pancreatic ductal adenocarcinoma	Low	Poor overall survival; reduced immunotherapy benefit	Tumor-suppressive	Enhances m^6^A on CDKN1A/p21, enforcing G1 arrest	34,60
Endometrial cancer	Low	Poor overall survival; advanced stage and immune checkpoint resistance	Tumor-suppressive	Low expression linked to immune checkpoint resistance (mechanism unresolved)	61,62
Epithelial ovarian cancer	Low	Poor disease-free survival; platinum chemoresistance	Tumor-suppressive	SNP rs11869256 reduces METTL16, affects platinum response	63
Papillary thyroid carcinoma	Low	Poor overall survival; early-stage tumor progression	Tumor-suppressive	DNMT1-mediated promoter hypermethylation lowers m^6^A on SCD1, altering lipid metabolism	15,64
Urothelial carcinoma	Low	Poor overall survival; cisplatin resistance	Tumor-suppressive	Stabilizes PMEPA1, and promotes autophagy and drug sensitivity	65

METTL16 expression is also closely related to tumor staging, grading, and metastatic status, providing valuable insights into its prognostic potential for specific cancer types. In esophageal cancer, METTL16 expression is positively correlated with clinical stage, with higher levels observed in patients at more advanced stages, suggesting its involvement in esophageal cancer progression, particularly in later stages.^48^ In breast cancer, METTL16 expression correlates with estrogen receptor (ER) and progesterone receptor (PR) levels, indicating that it may play different roles in various breast cancer subtypes.^56^ Furthermore, METTL16 expression is associated with clinical characteristics in glioblastoma, such as isocitrate dehydrogenase status, molecular subtype, and treatment response.^53^ These findings suggest that METTL16 may serve as a predictive biomarker for treatment response and resistance, aiding clinicians in tailoring personalized therapeutic strategies based on METTL16 status.

In breast cancer, METTL16 expression is closely linked to recurrence-free survival, highlighting its potential as a biomarker for predicting treatment efficacy.^56^ Additionally, studies have shown that METTL16 prevents ferroptosis by inhibiting lipid peroxidation and reactive oxygen species accumulation, which may help overcome resistance to conventional therapies. Inducing ferroptosis by inhibiting METTL16 could therefore address treatment resistance in cancer patients.^57^ Furthermore, research indicates that alterations in m^6^A regulatory factors, including METTL16, may influence treatment responses in pancreatic cancer. A novel patient stratification method based on the genetic status of METTL16 and other m^6^A regulators may help identify patients who are more sensitive or resistant to specific treatments.^66^

In conclusion, METTL16 plays a dual role in cancer, with its prognostic significance depending on the expression level and alterations in specific tumors, subtypes, and even individual patients. Understanding these nuances is essential for leveraging METTL16 as a potential biomarker in clinical practice.

### Impact of METTL16 regulation, protein modifications, and mutations on cancer development

The activity and function of METTL16 protein can be influenced by various translational modifications, post-translational modifications, and genetic mutations, which in turn impact cancer development and progression. Understanding the effects of these alterations is essenRNAtial for elucidating the role of METTL16 in tumorigenesis and identifying potential therapeutic targets.

#### Post-translational modifications of METTL16 protein

**Figure 2:**
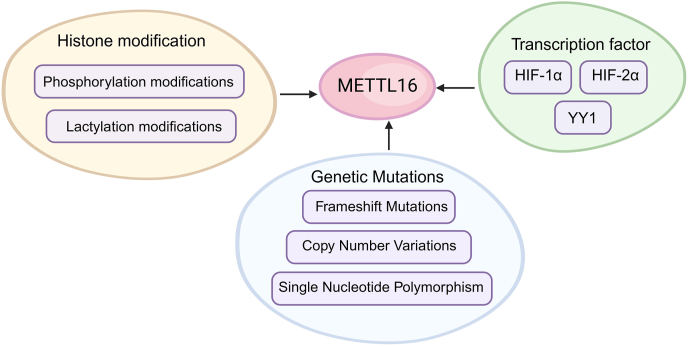
Regulation of METTL16 expression and function. The upstream regulatory region of METTL16 is modulated by various histone modifications, such as phosphorylation and lactylation, which can indirectly influence its gene expression and functional activity. Key transcription factors, including HIF-1α and HIF-2α, regulate METTL16 expression, particularly under hypoxic conditions. Additionally, the transcription factor YY1 plays a crucial role in the direct regulation of METTL16. Genetic alterations within the METTL16 gene, including frameshift mutations, copy number variations (CNVs), and single-nucleotide polymorphisms (SNPs), also affect its expression and functional state, contributing to its involvement in cancer progression and other diseases.

Phosphorylation of METTL16 at specific sites modulates its enzymatic activity and substrate specificity, thereby regulating the m^6^A methylation levels of target RNAs. Aberrant phosphorylation of METTL16 can disrupt RNA methylation patterns in cancer cells, influencing tumor growth and metastasis (Fig. 2). In pancreatic ductal adenocarcinoma, METTL16 phosphorylation plays a critical role in DNA damage repair, particularly in homologous recombination repair. METTL16 interacts with meiotic recombination 11 (MRE11) through RNA, inhibiting MRE11 exonuclease activity and preventing DNA end resection, a key early step in HR repair. Following DNA damage, the ataxia-telangiectasia mutated (ATM) kinase phosphorylates METTL16 at Ser419, triggering conformational changes that reduce its RNA-binding capability. This alteration relieves the inhibition on MRE11, facilitating DNA end resection and subsequent HR repair. This phosphorylation-dependent regulatory mechanism underscores METTL16's dual role in maintaining genome stability and its potential as a therapeutic target in HR repair-deficient cancers, including pancreatic ductal adenocarcinoma.^60^

Lactylation also affects METTL16's activity and stability, modulating cancer cell responses to environmental stimuli. In gastric cancer cells, elevated copper levels promote lactylation of METTL16 at the K229 site, mediated by enhanced interaction with acetyltransferases alanyl-tRNA synthetase 1/2 (AARS1/AARS2) (Fig. 2). This modification enhances METTL16's m^6^A methylation activity on FDX1 mRNA, increasing FDX1 protein expression, which is crucial for inducing cuproptosis (copper-induced cell death). Additionally, sirtuin 2 (SIRT2), as a deacetylase, suppresses lactylation at the K229 site, thereby regulating METTL16's activity. Lactylation at K229 disrupts METTL16's autoinhibitory conformation by altering salt–bridge interactions between key residues. This conformational change enhances the enzyme's methyltransferase activity, promoting m^6^A modifications of target RNAs. These changes significantly impact critical cellular processes, including regulating copper metabolism and promoting cuproptosis under elevated copper concentrations or other environmental stress conditions.^44^

#### Genetic mutations of METTL16

Recent studies have underscored the impact of METTL16 gene mutations in the development and progression of cancer, revealing several critical genetic alterations. Frameshift mutations are particularly common in colorectal cancer with high microsatellite instability, leading to dysregulated RNA methylation and altered cancer cell phenotypes (Fig. 2).^67^, ^68^, ^69^ In colorectal cancer with high microsatellite instability, inactivating mutations in methyltransferase genes like METTL16 disrupt RNA methylation, resulting in the loss of protein function. However, frameshift mutations in the METTL16 transcript that generate premature termination codons can still trigger nonsense-mediated decay, thereby compromising RNA stability.^68^

Additionally, copy number variations (CNVs) of the METTL16 gene are observed across various cancer types, notably in bladder cancer patients with TP53 mutations, suggesting a potential synergy in cancer transformation.^70^^,^^71^ In hepatocellular carcinoma, studies have linked CNV changes in genes such as KIAA1429 (also known as vir-like m^6^A methyltransferase-associated protein (VIRMA)) and METTL16 to hepatocellular carcinoma development, progression, and clinical prognosis. Specifically, the loss of METTL16 copy number is significantly associated with poor disease-free survival and has been identified as an independent risk factor.^72^ These findings indicate that CNV alterations may serve as valuable biomarkers for clinical diagnosis and prognosis, opening new avenues for personalized treatment (Fig. 2).

Moreover, the single-nucleotide polymorphism rs11869256 A > G on chromosome 17 is linked to a reduced risk of epithelial ovarian cancer, with the METTL16 rs11869256 GA genotype influencing gene expression regulation and clinical outcomes (Fig. 2).^63^ These mutations are also associated with altered sensitivities to anti-cancer drugs, highlighting their potential as predictive biomarkers for therapeutic responses.^64^

#### Transcriptional regulation of METTL16

In the tumor microenvironment, rapid tumor growth coupled with insufficient blood supply often leads to local hypoxia. This hypoxic condition triggers a series of adaptive responses, including the activation of hypoxia-inducible factors (HIFs). HIFs are a family of transcription factors that respond to hypoxia by regulating the expression of various genes through direct binding to hypoxia-response elements (HREs) in the promoter regions of target genes.^73^ Under hypoxic conditions, the stability and activity of HIF-1α are enhanced. HIF-1α binds to the HRE in the promoter region of METTL16, thereby up-regulating its expression at the transcriptional level (Fig. 2).^42^ Conversely, HIF-2α exerts a direct inhibitory effect on METTL16 expression. By binding to the HRE sequence in the METTL16 promoter, HIF-2α inhibits its transcriptional activity, potentially affecting the proliferative capacity of bladder cancer cells and their sensitivity to chemotherapy (Fig. 2).^65^

Furthermore, studies have shown that Yin Yang 1 (YY1) regulates METTL16 expression by binding to specific sites in its promoter region. YY1 suppresses METTL16 transcription, thereby modulating its expression (Fig. 2).^49^ These transcriptional regulatory mechanisms are crucial for the functional state of cells and tumor progression, particularly in the context of METTL16's role in cancer development.

## Mechanistic basis of METTL16-mediated RNA metabolism in cancer

### m^6^A-dependent mechanisms in mRNA splicing, stability, and turnover

Recent research has highlighted the methyltransferase activity of METTL16, underscoring its significant role in regulating mRNA stability and splice site selection. While the number of confirmed direct methylation targets for METTL16 remains limited, its absence leads to a substantial reduction in m^6^A modifications across the genome, suggesting a broad impact on the epitranscriptome.^11^^,^^74^ As a key m^6^A methyltransferase, METTL16 plays a central role in RNA methylation, primarily adding m^6^A marks to newly synthesized RNA, particularly at introns. Notably, the m^6^A modifications on introns are associated with polyadenylation processes. Across multiple models, METTL16-mediated intronic m^6^A sites are associated with the selection of alternative polyadenylation sites. These events are frequently dysregulated in cancer, leading to the production of non-coding transcripts or truncated proteins. These findings suggest that METTL16's m^6^A marking may have significant implications in cancer biology.^75^

**Figure 3:**
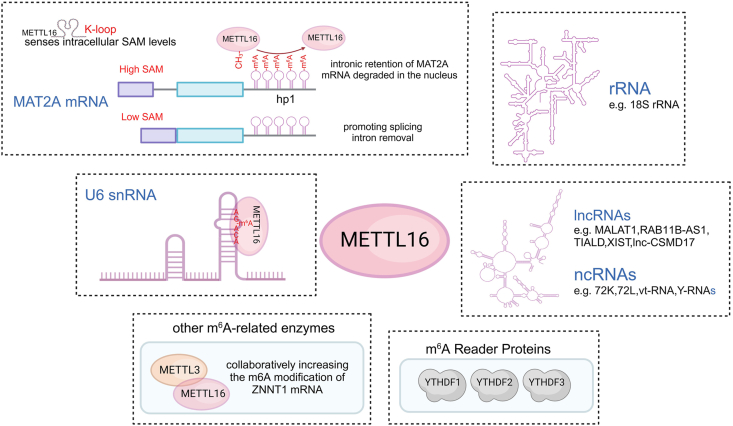
Various targets of METTL16. METTL16 senses intracellular SAM levels through its K-loop structure and regulates MAT2A splicing accordingly (see the subsection “m^6^A-dependent mechanisms in mRNA regulation” for details). Furthermore, METTL16 interacts with rRNA (ribosomal RNA, such as 18S rRNA) and other non-coding RNAs (*e.g.*, lncRNAs and ncRNAs, including MALAT1, XIST, and Y-RNAs), participating in the post-transcriptional regulation of various RNAs. In collaboration with other m^6^A-related enzymes, METTL16 can work with METTL3 to enhance the m^6^A modification of ZNNT1 mRNA. Meanwhile, METTL16, through its collaboration with m^6^A reader proteins (such as YTHDF1, YTHDF2, and YTHDF3), recognizes and binds to m^6^A-modified RNA, regulating the local structure of RNA, its interactions with other molecules, and its cellular fate.

One of the earliest and most extensively studied targets of METTL16 is MAT2A mRNA, which encodes a key enzyme that synthesizes the methyl donor SAM. METTL16 regulates the biosynthesis of SAM by promoting either mRNA degradation or the proper splicing of mRNA. This regulatory mechanism plays a crucial role in maintaining the metabolic balance of the cell (Fig. 3).^11^^,^^18^ METTL16's regulation of SAM levels is essential for cellular methylation reactions, which affect DNA, RNA, and protein methylation. The unique K-loop mechanism of METTL16 enables it to sense intracellular SAM levels, modulating its activity accordingly. Under high SAM concentrations, METTL16 methylates MAT2A mRNA at a conserved site (hp1) in the 3′ UTR, leading to rapid dissociation from the RNA, retention of introns, and reduced MAT2A protein synthesis, thereby preventing excessive SAM accumulation. In contrast, when SAM levels are low, the catalytic efficiency of METTL16 decreases, leading to a prolonged binding duration with hp1. This extended binding induces proper splicing of MAT2A mRNA, removing introns and generating mature mRNA, which in turn restores SAM levels.^6^^,^^11^^,^^29^ The cleavage factor Im (CFIm) complex, a key splicing factor, also plays a pivotal role in MAT2A mRNA processing. Studies have shown that CFIm25 also regulates MAT2A splicing. In high SAM conditions, the CFIm complex alters splicing efficiency, resulting in intron retention and lower MAT2A protein levels, thus regulating SAM synthesis.^76^ However, the precise regulatory relationship between CFIm25 and METTL16 in maintaining SAM homeostasis remains unclear. In cancer, where cellular methionine dependency is often elevated, METTL16's regulation of SAM levels through MAT2A mRNA splicing becomes particularly important.^8^^,^^77^ By controlling MAT2A splicing and stability, METTL16 ensures that SAM levels remain balanced, which is essential for tumor cell survival and proliferation. These insights into METTL16's role in SAM metabolism highlight its relevance in cancer biology and underscore its potential as a therapeutic target.

Within the broader m^6^A regulatory network, METTL16 interacts with other methyltransferases, such as METTL3 and METTL14, as well as demethylases like fat mass and obesity-related associated protein (FTO) and alkylation repair homolog protein 5 (ALKBH5), creating a complex system that modulates gene expression through dynamic m^6^A modifications.^12^ In hepatocellular carcinoma, METTL16 and METTL3 collaborate to enhance m^6^A modifications on ZNF706 neighboring transcript 1 (ZNNT1) mRNA, thereby increasing its stability and expression. This interaction not only drives tumor cell proliferation and invasion but also contributes to macrophage polarization in the tumor microenvironment, further promoting hepatocellular carcinoma progression.^78^ Additionally, METTL16 interacts with m^6^A “reader” proteins, such as members of the YTH N6-methyladenosine RNA binding protein F (YTHDF) family, to regulate RNA function. These reader proteins recognize m^6^A-modified RNAs, influencing their structure, interactions with other biomolecules, and cellular fate, including splicing, translation, nuclear export, and degradation (Fig. 3).^72^^,^^79^ In this way, METTL16 indirectly impacts RNA function and cellular processes through m^6^A modification. The critical role of METTL16 in regulating methyl donor capacity in mammals underscores its essential functions in both the methylome and transcriptome. Studies in mouse models show that METTL16 is crucial for development and survival, with its catalytic and RNA-binding activities being indispensable for these processes.^18^^,^^26^ A knock-in mouse model has further demonstrated that METTL16's methyltransferase and RNA-binding functions are vital for proper organismal development.^18^^,^^28^

METTL16 regulates MAT2A splicing and stability to maintain intracellular SAM levels. It collaborates with other m^6^A-related molecules to influence tumor cell proliferation, invasion, and the shaping of the tumor microenvironment, playing a critical role in tumor initiation and progression. Advancing our understanding of METTL16's mechanisms not only deepens insights into the significance of RNA modifications in tumor metabolic reprogramming and epigenetic regulation but also suggests novel avenues for its potential use as a therapeutic target or molecular diagnostic marker.

### m^6^A-independent regulation of mRNA stability and translation

**Figure 4:**
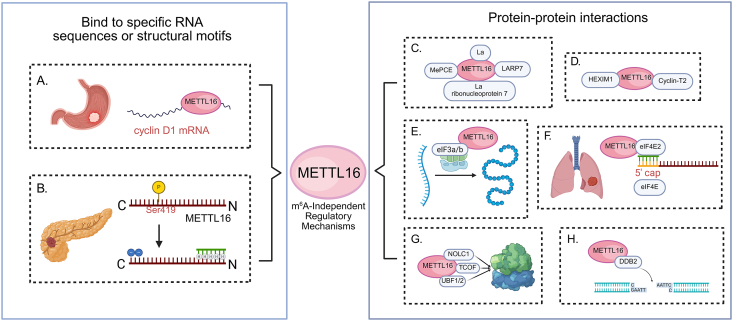
m^6^A-independent regulation of METTL16 in cancer. **(A)** In gastric cancer, METTL16 binds to specific sequences on cyclin D1 mRNA, influencing cell cycle progression and promoting tumor cell proliferation. **(B)** The binding of METTL16 to key RNAs, including those involved in pancreatic-associated RNA binding, is modulated by phosphorylation at Ser419, highlighting the role of post-translational modifications in its regulatory function. **(C)** Moreover, in RNA processing, METTL16 interacts with early U6 ribonucleoprotein assembly factors, such as MePCE and LARP7, in an RNA-dependent manner, contributing to the assembly and functional regulation of the 7SK snRNP complex. **(D)** This interaction modulates the processing and stability of 7SK RNA via HEXIM1 and cyclin T2. **(E)** Additionally, METTL16 directly interacts with translation initiation factors (*e.g.*, eIF3a/b), facilitating efficient translation of target transcripts. **(F)** In translation regulation, METTL16 inhibits eIF4E2 activity and enhances the recognition of the mRNA 5′ cap structure by eIF4E, thus promoting protein synthesis. **(G)** In regulating processes in the nucleolus, METTL16 associates with nucleolar proteins such as NOLC1, TCOF, and UBF1/2, regulating rRNA synthesis and processing. **(H)** METTL16 also participates in DNA damage repair by interacting with the nucleotide excision repair protein DDB2.

METTL16 exerts regulatory functions in cancer through m^6^A-independent mechanisms, involving direct interactions with RNA transcripts or proteins. Unlike other m^6^A writers such as METTL3 and METTL14, which form stable protein complexes, METTL16 operates independently, influencing gene expression through RNA interactions rather than requiring the presence of other core subunits. METTL16 can bind to specific RNA sequences or structural motifs independent of m^6^A modification, thereby regulating RNA stability, localization, or translation efficiency. For instance, in gastric cancer, METTL16 binds to cyclin D1 mRNA, influencing its stability and expression, which in turn promotes cell cycle progression and cellular proliferation (Fig. 4A).^45^ In pancreatic ductal adenocarcinoma, METTL16 regulates MRE11 nuclease activity independent of its methyltransferase function, as detailed in the subsection “post-translational modifications of METTL16 protein” (Fig. 4B).^60^

METTL16 does not form a stable protein complex like METTL3/14 and instead participates in dynamic protein–protein interactions, mediated by RNA or direct protein–protein contacts.^18^^,^^35^^,^^80^ These interactions enable METTL16 to engage with proteins involved in RNA metabolism, cellular signaling, and DNA repair, thereby modulating its methyltransferase activity and localization in response to cellular stresses, which is critical for cancer cell adaptation. In RNA processing, METTL16 binds to the early U6 biogenesis factors La, La ribonucleoprotein domain family member 7 (LARP7), and methylphosphate capping enzyme (MePCE) (Fig. 4C), facilitating m^6^A installation on U6 snRNA and its maturation. It also associates with the 7SK snRNP regulators hexamethylene bisacetamide-inducible protein 1 (HEXIM1) and cyclin T2, influencing 7SK RNA function (Fig. 4D).^22^^,^^35^ This interaction facilitates METTL16's role in the methylation of U6 snRNA, influencing its processing and maturation. METTL16's involvement in translation is equally significant. In the cytoplasm, it promotes translation by recruiting eIF3a/b and rRNA in an RNA-independent manner, increasing translation efficiency, particularly in cancer-related pathways (Fig. 4E).^9^^,^^26^ METTL16 directly interacts with rRNAs, including 18S and 28S–5.8S rRNA, enhancing the assembly of the 43S pre-initiation complex (PIC) and 80S translation initiation complex (TIC), which is crucial for the translation of mRNAs involved in tumorigenesis.^81^ In lung cancer, METTL16 has been shown to interact with eIF4E2, a translation repressor, preventing its interaction with the mRNA 5′ cap structure. This interaction promotes eIF4E recognition of the cap, thereby enhancing translation efficiency (Fig. 4F). Notably, knockout of METTL16 reduces the translation efficiency of oncogenes such as procollagen-lysine,2-oxoglutarate 5-dioxygenase 2 (PLOD2), while knockout of eIF4E2 has the opposite effect, demonstrating the critical role of METTL16 in selectively enhancing the translation of oncogenic proteins.^32^ Within the nucleolus, METTL16 partners with treacle ribosome biogenesis factor (TCOF), nucleolar and coiled-body phosphoprotein 1 (NOLC1), and upstream binding factor 1/2 (UBF1/2) to promote rRNA synthesis and processing (Fig. 4G).^81^ METTL16 also interacts with damage-specific DNA-binding protein 2 (DDB2), a protein involved in nucleotide excision repair, indicating its potential role in DNA repair mechanisms, especially in response to DNA damage (Fig. 4H).^81^

The role of METTL16 in tumor cells extends beyond m^6^A modification-dependent mechanisms. It dynamically interacts with various mRNAs, proteins, and RNA-protein complexes, influencing key cancer-related pathways such as cell cycle progression, DNA damage repair, and translational regulation. The versatility and adaptability of METTL16 in different tumors underscore its central role in cancer cell adaptation and survival. By delving deeper into its functions within tumor metabolism and transcriptional and post-transcriptional regulatory networks, we may identify critical nodes in cancer initiation and progression, paving the way for breakthroughs in precision medicine and personalized therapeutic strategies.

### Regulation of non-coding RNAs

Recent advances in the identification of RNA targets interacting with METTL16 have revealed a broad spectrum of potential m^6^A-modified substrates, including both coding and non-coding RNAs. Although hundreds of direct candidate targets have been identified, many of these still require further validation. METTL16 is primarily recognized for its role in catalyzing m^6^A modification on U6 snRNA. By introducing m^6^A modifications at specific sites, METTL16 can influence the structure and function of U6 snRNA, thereby impacting precursor mRNA processing. U6 snRNA is a key component of the spliceosome, where it plays a central role in positioning substrates for splicing reactions.^82^^,^^83^

MePCE adds a methylated cap structure to the 5′ end of U6 snRNA, protecting it from degradation and stabilizing its function.^84^ La protein, an RNA chaperone, binds to newly synthesized U6 snRNA, assisting in proper folding and preventing its degradation.^85^ LARP7, another key binding protein of U6 snRNA, binds to the oligouridine sequence at the 3′ end, stabilizing its structure and enhancing its assembly and function within the spliceosome, thus supporting U6 snRNA biogenesis.^86^^,^^87^ METTL16 interacts with MePCE, La protein, and LARP7 via an RNA-dependent mechanism during the early processing stages of U6 snRNA. Together, these proteins stabilize the 5′-end methylation, 3′-end oligouridine modifications, and overall RNA folding structure of U6 snRNA, promoting its maturation. m^6^A modifications, such as those deposited by METTL16, regulate the stability of U6 snRNA and its interactions with both RNA and proteins. Specifically, METTL16 deposits an m^6^A modification on a protrusion in the stem of the hairpin structure of human U6 snRNA, known as the ACAGA box.^11^^,^^18^^,^^22^

This sequence is part of an evolutionarily conserved region that pairs with the 5′ splice site of pre-mRNA, playing a crucial role in the first step of splicing.^10^^,^^88^^,^^89^ The A43 site within the highly conserved ACm6AGAGA sequence directly participates in the base pairing between U6 snRNA and the 5′ splice site of precursor mRNA.^90^ The m^6^A modification at the A43 site of U6 snRNA not only alters the RNA's stability and its interaction with precursor mRNA but also significantly impacts the assembly of the spliceosome and the recognition of the 5′ splice site, thus exerting broader effects on precursor mRNA splicing (Fig. 3). Structural studies indicate that this m^6^A mark alters the local RNA secondary structure, enhancing the dynamic interactions between U6 snRNA and spliceosome components, optimizing the catalytic efficiency of the splicing machinery. Moreover, the m^6^A modification at position 43 mediated by METTL16 regulates splicing outcomes for nearly all types of precursor mRNA (both coding and non-coding RNAs), integrating into the fine-tuned regulation of gene expression. This regulation of the epitranscriptome underscores the importance of U6 snRNA, as a non-coding RNA, in the precise assembly of the spliceosome and the shaping of the transcriptome landscape.^22^^,^^91^^,^^92^ Consequently, although METTL16-catalyzed m^6^A modification primarily affects non-coding U6 snRNA, its downstream effects extend far beyond non-coding RNAs, influencing gene expression and RNA processing within the cell. These extensive regulatory actions highlight METTL16 as a significant factor in cellular function and disease progression.^22^^,^^93^

In addition to its role in modulating U6 snRNA, METTL16 regulates the expression and function of non-coding RNAs (ncRNAs), long non-coding RNAs (lncRNAs), and ribosomal RNA (rRNA) in cancer.^22^^,^^74^^,^^79^^,^^94^ These regulatory interactions contribute to diverse cellular processes implicated in tumorigenesis. The interaction between METTL16 and 7SK and 7SL RNA demonstrates the role of this methyltransferase in regulating the function of ncRNAs (Fig. 3). For 7SK RNA, METTL16 participates in its methylation by interacting with components of the 7SK small nuclear ribonucleoprotein (snRNP), such as LARP7 and MePCE. This interaction may affect the assembly and function of the 7SK snRNP, thereby influencing RNA polymerase II activity and significantly affecting the transcription process. For 7SL RNA, METTL16 interacts with SRP14, a component of the signal recognition particle (SRP), which is critical for protein secretion and localization.^22^ Additionally, some ncRNAs, such as Vault RNA and Y RNA, have been found to interact with METTL16, potentially affecting their structure, stability, and intracellular functions. However, further experimental work is needed to clarify the specific molecular mechanisms and biological consequences of these interactions.^35^

METTL16-mediated modifications or interactions may affect the stability, localization, or activity of oncogenic or tumor-suppressive lncRNAs, thereby influencing gene expression programs and cellular phenotypes associated with cancer progression (Fig. 3). Notably, the long non-coding RNA metastasis-associated lung adenocarcinoma transcript 1 (MALAT1) recruits METTL16 to the hairpin-1 region of MAT2A mRNA, thereby linking SAM availability to MAT2A translation; the molecular details of this feedback sensor are described in the subsection “m^6^A-dependent mechanisms in mRNA regulation”.^95^ Additionally, METTL16 can directly interact with the long non-coding RNA RAB11B antisense RNA 1 (RAB11B-AS1), inducing its m^6^A modification and reducing the RNA stability of RAB11B-AS1, leading to decreased expression and affecting hepatocellular carcinoma progression.^47^ Studies also suggest that in hepatocellular carcinoma, the lncRNA transcript inducer of AURKA lysosomal degradation (TIALD) acts as a tumor suppressor. Loss of METTL16 increases TIALD stability, while knockdown of m^6^A readers YTHDF2 and YTHDC1 further enhances TIALD stability, contributing to the suppression of metastasis. Conversely, overexpression of ALKBH5 (an m^6^A eraser) significantly enhances TIALD stability, promoting metastasis.^96^ Furthermore, lncRNAs such as X inactive specific transcript (XIST) and lnc-CSMD1-7 have been shown to interact with METTL16, and these interactions may influence cancer metastasis and poor prognosis.^42^^,^^97^

The human m^6^A modifications on rRNA also affect selective ribosomal translation under cellular stress.^98^^,^^99^ METTL16 interacts with several key proteins in the nucleolus, which may be involved in the regulation of rRNA synthesis and modification (Fig. 3). It may regulate the synthesis of 18S rRNA through m^6^A modifications, which are closely associated with ribosomal biogenesis.^81^ METTL16 acts directly on rRNA in liver cancer stem cells and promotes its maturation. Through this mechanism, METTL16 facilitates the assembly and biosynthesis of ribosomes, thereby increasing the formation of fully functional ribosomes within the cell.^46^

METTL16 exerts profound effects on core processes in tumor cells by regulating a multilayered RNA network, including U6 snRNA, ncRNA, lncRNA, and rRNA. These processes encompass post-transcriptional regulation, alternative splicing, and ribosome assembly. Ongoing research into the molecular mechanisms of METTL16 may enable targeted interventions in its regulatory pathways, opening new possibilities for precision cancer therapy and prognostic evaluation. A curated list of experimentally confirmed METTL16 RNA substrates, together with the assay used and functional read-out, is provided in Table 3.

**Table 3:** Experimentally validated direct RNA targets of METTL16.

RNA type	Cancer model/cell line	Validation method	Functional outcome	Reference
MAT2A mRNA	Hepatocellular carcinoma, pancreatic ductal adenocarcinoma cells	miCLIP; site-direct mutagenesis	Controls intron retention; tunes SAM levels	11
U6 snRNA	Universal	miCLIP; SCARLET	Modulates 5′ splice-site recognition	11
BCAT1/BCAT2 mRNAs	Acute myeloid leukemia cell lines; patient-derived xenograft	MeRIP-seq; mut rescue	Promotes BCAA catabolism and leukaemia stem cell maintenance	36
SOGA1 mRNA	Colorectal cancer cells	RIP-qPCR; MeRIP-qPCR	Stabilizes SOGA1, activates PDK4, enhances glycolysis	^100^
FDX1 mRNA	Gastric cancer cells	MeRIP-qPCR; ActD chase	Up-regulates FDX1, sensitises cuproptosis	44
GPX4 mRNA	Breast cancer	MeRIP-qPCR; luciferase reporter	Increases GPX4 translation and ferroptosis resistance	57
CDKN1A mRNA	Pancreatic ductal adenocarcinoma cells	ActD-chase; MeRIP-qPCR	Elevates p21 protein, enforces G1 arrest	^101^
RAB11B-AS1 lncRNA	Hepatocellular carcinoma	MeRIP-qPCR; RNA-decay assay	Reduces lncRNA stability, limits tumor growth	^102^

Note: miCLIP, methylation-individual nucleotide resolution cross-linking and immunoprecipitation; SCARLET, site-specific cleavage and radio-labeling followed by thin-layer chromatography; RIP-qPCR, RNA immunoprecipitation followed by quantitative PCR; MeRIP-qPCR, methylated RNA immunoprecipitation followed by qPCR; ActD chase, actinomycin D transcriptional shut-off (chase) assay.

## Molecular mechanisms of METTL16 in tumorigenesis

Building on these direct substrates, we next examine how METTL16 integrates into major oncogenic pathways. METTL16 plays a crucial role in regulating key oncogenic signaling pathways and metabolic processes associated with cancer development and progression. By modulating the methylation status of specific RNAs, METTL16 affects the expression and activity of various oncogenes and tumor suppressor genes. This regulatory effect extends to key signaling pathways that are essential for cell proliferation, survival, and differentiation (Table 4).

**Table 4:** Molecular mechanisms of METTL16 in tumorigenesis.

Cancer type	Target gene	Signaling pathway	Whether it depends on m^6^A modification	Role of METTL16	Mechanism	References
Pancreatic ductal adenocarcinoma	DVL2	Wnt/β-catenin pathway	Yes	Negatively regulates	Catalyzes the m^6^A modification on DVL2 mRNA and inhibits its translation	^103^
Pancreatic cancer	CDKN1A (p21)	p21 signaling pathway	Yes	Positively regulates	Delays the G1 phase and enhances CDKN1A mRNA stability	^101^
Breast cancer	GPX4	Ferroptosis pathway	Yes	Negatively regulates	Proposed to enhance GPX4 pre-mRNA splicing/translation	57
Non-small cell lung cancer	GCN2	GCN2-ATF4 signaling pathway	No	Positively regulates	Directly suppresses protein translation or indirectly decreases GCN2-ATF4-mediated transcription	^104^
Bladder cancer	PMEPA1	Autophagy pathway	Yes	Negatively regulates	Decreases PMEPA1 mRNA stability	65
Hepatocellular carcinoma	SENP3	Ferroptosis pathway	Yes	Negatively regulates	Increases SENP3 RNA stability	41
Hepatocellular carcinoma	lnc-CSMD1-7	Hypoxia pathway	Yes	Negatively regulates	Decreases lnc-CSMD1-7 RNA stability	42
Papillary thyroid carcinoma	SCD1	Lipid metabolism	Yes	Positively regulates	Decreases SCD1 mRNA stability	15
Colorectal cancer	SOGA1, PDK4	Glycolysis	No	Positively regulates	Enhances SOGA1 mRNA stability and activates PDK4	^100^
Acute myeloid leukemia	BCAT1, BCAT2	Branched-chain amino acid metabolism	Yes	Positively regulates	Decreases m^6^A abundance and the stability of BCAT1/2 mRNAs	36
Gastric cancer	FDX1	Cuproptosis	Yes	Positively regulates	Enhances FDX1 mRNA stability	44

### Wnt/β-catenin pathway

METTL16 has been implicated in the regulation of the Wnt/β-catenin signaling pathway, a critical pathway involved in cancer initiation, progression, and metastasis. Dysregulation of METTL16-mediated RNA modifications can alter the expression of key components of the Wnt pathway, leading to aberrant signaling and contributing to tumorigenesis. For example, METTL16 regulates the m^6^A modification of dishevelled 2 (DVL2) mRNA, a key component of the Wnt/β-catenin signaling pathway. The down-regulation of METTL16 leads to a concomitant increase in DVL2 levels, thereby promoting the progression of pancreatic ductal adenocarcinoma.^103^^,^^105^

### p21 signaling pathway

The p21 signaling pathway plays a crucial role in regulating the cell cycle and apoptosis. As an inhibitor of cyclin-dependent kinases, p21 can halt the cell cycle in response to DNA damage, thus preventing the propagation of genetic errors. Dysregulation of p21 expression is associated with uncontrolled cell division, reduced apoptosis, and increased tumor aggressiveness.^106^ In pancreatic cancer, down-regulation of METTL16 leads to decreased m^6^A modification of cyclin-dependent kinase inhibitor 1A (CDKN1A, also known as p21) mRNA, which in turn reduces the expression of the p21 protein. This reduction in p21 levels facilitates the progression of the cell cycle from the G1 phase to the S phase, promoting cell proliferation. On the other hand, overexpression of METTL16 enhances the m^6^A modification of CDKN1A, increasing p21 expression. This results in the arrest of the cell cycle at the G1 phase, inhibiting tumor cell proliferation and suggesting a potential therapeutic strategy for controlling tumor growth.^101^

### Ferroptosis

Ferroptosis is a form of programmed cell death characterized by the accumulation of lipid peroxides to lethal levels.^107^^,^^108^, ^109^ In hepatocellular carcinoma, METTL16 enhances the expression of SENP3 mRNA by catalyzing its m^6^A modification. This, in turn, increases the stability of lactoferrin (LTF) through the desumoylation activity of SENP3. The enhanced LTF then chelates free iron ions, reducing the labile iron pool and thereby decreasing the occurrence of ferroptosis. In this way, METTL16 plays a critical regulatory role in ferroptosis in hepatocellular carcinoma by modulating the expression and function of SENP3 and LTF.^41^ Glutathione peroxidase 4 (GPX4) is another key player in ferroptosis. It catalyzes the conversion of toxic lipid peroxides into non-toxic lipid alcohols, counteracting ferroptosis.^110^, ^111^, ^112^ As a central enzyme in cellular antioxidant defenses, GPX4 is essential for preventing ferroptosis.^113^ In breast cancer, the up-regulation of METTL16 expression leads to increased m^6^A modification of GPX4 mRNA, enhancing the expression of GPX4. This METTL16-mediated regulation helps breast cancer cells evade ferroptosis, promoting cancer growth and development.^57^

### GCN2-ATF4 signaling pathway

The general control nonderepessible 2 (GCN2)-activating transcription factor 4 (ATF4) signaling pathway plays a pivotal role in cellular responses to amino acid deprivation and stress. Disruption of this pathway can impair the cell's ability to adapt to nutrient scarcity, promoting the progression of metabolic diseases and facilitating tumor survival under nutrient-poor conditions. In non-small cell lung cancer, under amino acid restriction conditions, the loss of METTL16 reduces GCN2 activity, leading to decreased phosphorylation of eIF2α. This reduction in phosphorylation inhibits the synthesis of ATF4 protein. The lowered expression of ATF4 affects the promoter activity of apoptosis-related genes such as ATF3, CCAAT/enhancer-binding protein (C/EBP) homologous protein (CHOP), and death receptor 5 (DR5). This disruption weakens the cellular stress response to nutrient deprivation, thereby enhancing the survival of cancer cells within the nutrient-deficient tumor microenvironment.^104^

### Autophagy

Autophagy is a vital cellular process responsible for the degradation and recycling of damaged or unnecessary components, helping to maintain cellular homeostasis and energy balance, particularly under stress conditions. Dysregulation of autophagy can influence disease progression, either by promoting the survival of stressed cells or by contributing to cell death.^114^ METTL16, through its m^6^A methyltransferase activity, decreases the stability of prostate transmembrane androgen inducible protein 1 (PMEPA1) mRNA, leading to reduced expression of the PMEPA1 protein. PMEPA1 co-localizes with lysosomes and late endosomes, playing a crucial role in promoting autophagy. This down-regulation of PMEPA1 inhibits the proliferation of bladder cancer cells and enhances their sensitivity to cisplatin, highlighting the potential of targeting METTL16 in cancer therapy.^65^

### Hypoxia pathway

The hypoxia pathway is activated under low-oxygen conditions, primarily regulated by the stabilization of HIFs. These factors drive the expression of genes that help cells adapt to hypoxic conditions, including those involved in angiogenesis, metabolism, and survival. Abnormal activation of the hypoxia pathway is a hallmark of many cancers, promoting tumor growth, metastasis, and resistance to chemotherapy by enabling tumor cells to thrive in the hypoxic tumor microenvironment.^102^ Under low-oxygen conditions, the expression of METTL16 can be up-regulated via the HIF-1α pathway. METTL16, through m^6^A methylation, reduces the RNA stability of lnc-CSMD1-7, which promotes the metastasis and progression of hepatocellular carcinoma. Additionally, lnc-CSMD1-7 interacts with the RNA splicing factor RNA binding fox-1 homolog 2 (RBFOX2), influencing its splicing activities and further modulating the expression of epithelial-mesenchymal transition-related genes. This signaling pathway plays a critical role in promoting metastasis and poor prognosis in the pathogenesis of hepatocellular carcinoma.^42^

### Lipid metabolism

Lipid metabolism encompasses the synthesis and degradation of lipids within cells, playing essential roles in energy storage, hormone production, and membrane structure. In cancer, dysregulated lipid metabolism, particularly the overactivity of fatty acid synthesis pathways such as fatty acid synthase (FASN), promotes rapid proliferation and survival of tumor cells under adverse conditions.^115^ In papillary thyroid carcinoma, DNA hypermethylation mediated by DNA methyltransferase 1 (DNMT1) in the promoter region of METTL16 leads to reduced transcription and expression of METTL16. The decreased expression of METTL16 results in a reduction of m^6^A modifications on stearoyl-CoA desaturase 1 (SCD1) mRNA, thereby inhibiting its degradation process and leading to increased SCD1 expression. This enhances lipid metabolism in papillary thyroid carcinoma cells, promoting tumor progression and contributing to poor clinical prognosis.^15^

### Glycolysis

Glycolysis is the metabolic pathway that converts glucose into pyruvate, generating small amounts of adenosine triphosphate (ATP) and reduced nicotinamide adenine dinucleotide (NADH). Cancer cells often exhibit enhanced glycolysis, a phenomenon known as the Warburg effect, which meets their increased energy demands for rapid growth and survival under aerobic conditions.^116^ In colorectal cancer, METTL16 plays a key role in promoting glycolytic metabolism and tumor progression by regulating the expression of key glycolytic enzymes, such as SOGA1 and pyruvate dehydrogenase kinase 4 (PDK4). METTL16 stabilizes SOGA1 mRNA, thereby increasing its expression. This, in turn, activates PDK4 through SOGA1, which further contributes to the glycolytic shift in cancer cells and supports their metabolic demands during tumorigenesis.^49^

### Branched-chain amino acid metabolism

The metabolic role of branched-chain amino acids (BCAAs) in cancer is crucial, as they provide essential nutrients for tumor cell growth, supporting protein synthesis and nucleic acid production. BCAA metabolism is closely linked to the rapid proliferation, invasion, and drug resistance of cancer cells, making it a promising target for therapeutic strategies.^117^ Through its m^6^A methyltransferase activity, METTL16 enhances the expression of the enzymes BCAT1 and BCAT2, which catalyze the conversion of BCAAs into their corresponding branched-chain α-keto acids (BCKAs). These metabolites are integral to energy metabolism and ribosomal biosynthesis, thereby supporting the growth and proliferation of leukemia cells. Moreover, METTL16 reprograms BCAA metabolism in acute myeloid leukemia cells by modulating the expression of these key enzymes. This reprogramming helps maintain the self-renewal capacity of leukemia stem cells and promotes cancer progression.^36^

### Cuproptosis

Cuproptosis, a unique copper-dependent mode of cell death, differs from other well-known forms of cell death and holds significant promise in cancer therapy. This process is closely associated with cellular energy metabolism, particularly mitochondrial respiration and the lipoic acid (LA) pathway, enabling copper to directly influence the survival and death of cancer cells. Moreover, copper ion carriers offer a potential avenue for targeted cancer treatment, where cuproptosis induction, combined with small molecule drugs, could be used to selectively treat certain cancer types.^118^, ^119^, ^120^ In gastric cancer, elevated copper ion levels lead to the lactylation of METTL16 at the K229 site, which enhances its interaction with acetyltransferases AARS1 and AARS2. This interaction promotes METTL16's m^6^A modification activity on FDX1 mRNA. FDX1, a key regulator of copper ion carrier-induced cell death, becomes up-regulated, directly triggering cuproptosis. Additionally, SIRT2, a deacetylase, can inhibit METTL16 acetylation, thereby indirectly regulating FDX1 expression and the activation of cuproptosis. This mechanism not only underscores the central role of METTL16 in regulating copper ion-induced cell death but also suggests a potential therapeutic strategy for cancers in high-copper environments, particularly in the treatment of gastric cancer. The combined use of copper ion carriers and specific SIRT2 inhibitors could effectively promote METTL16 acetylation, enhancing the therapeutic potential of copper death.^44^

## Regulation of tumor immunity

**Figure 5:**
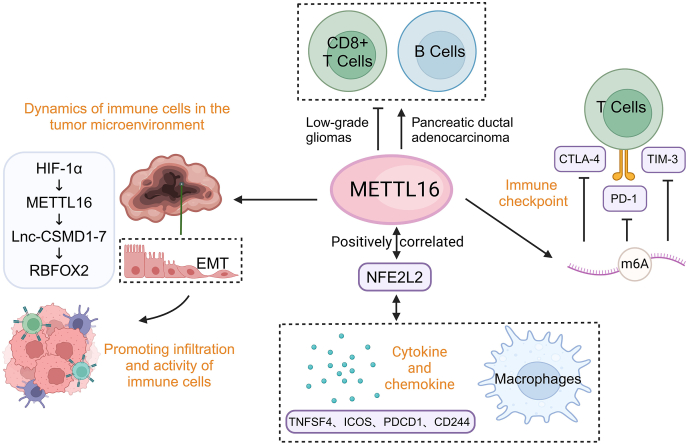
METTL16 in the regulation of tumor immunity. In the tumor microenvironment, METTL16 promotes epithelial–mesenchymal transition (EMT) by regulating the expression of lnc-CSMD1-7, thereby enhancing tumor invasion and immune cell activity. Additionally, METTL16 influences the expression of cytokines by regulating NFE2L2, affecting the release of chemokines (such as TNFSF4, ICOS, PDCD1, and CD244), which in turn regulates the function of macrophages and other immune cells. METTL16 also regulates the expression of T cell immune checkpoint molecules (CTLA-4, TIM-3, and PD-1) through its control of m^6^A modifications, thereby modulating tumor immune escape. Moreover, METTL16 regulates the activity of CD8^+^ T cells and B cells and their functions in tumor immunity, highlighting its multifaceted regulatory role in the tumor immune network.

Recent studies have shown that m^6^A modifications not only play an important role in regulating gene expression but also serve as a critical factor in immune modulation.^121^, ^122^, ^123^, ^124^, ^125^, ^126^ This finding offers a fresh perspective for understanding the mechanisms of immune regulation within the tumor microenvironment. METTL16 also modulates immune-related processes within the tumor microenvironment, influencing tumor immune evasion, inflammation, and anti-tumor immunity. Through RNA modifications or interactions, METTL16 may regulate the expression of key immune checkpoint molecules, such as programmed death-ligand 1 (PD-L1), cytotoxic T-lymphocyte-associated antigen-4 (CTLA-4), and T-cell immunoglobulin and mucin domain-containing protein 3 (TIM-3), as well as immune stimulators and major histocompatibility complex (MHC) molecules, thereby affecting tumor immune evasion and the response to immunotherapy^51^^,^^61^, ^127^ (Fig. 5). Overexpression of METTL16 has been shown to significantly reduce the proportion of PD-1-positive cells in activated T cells, suggesting that METTL16's regulatory activity may reduce immune evasion and enhance the immune system's ability to recognize and attack tumor cells.^51^ METTL16 can down-regulate PD-L1 mRNA levels via m^6^A modification, impacting the stability and degradation of PD-L1 mRNA. This regulation plays a crucial role in immune escape mechanisms at the cellular level. PD-L1 is a ligand protein produced by tumor cells that binds to PD-1 on T lymphocytes. This interaction suppresses immune recognition and reduces the cytotoxic activity of T lymphocytes against tumor cells. Furthermore, a colorectal cancer cell/T-cell co-culture system demonstrated that overexpression of METTL16 in colorectal cancer cells partially inhibits the proportion of PD-1-positive activated T cells. These findings suggest that by modulating PD-L1 expression, METTL16 can influence the response to immune checkpoint inhibitors, highlighting its potential as a therapeutic target to overcome resistance to immune checkpoint inhibitor therapy in patients with colorectal cancer.^51^^,^^128^ In gliomas, a strong positive correlation has been observed between METTL16 and nuclear factor erythroid-derived 2-like 2 (NFE2L2), with NFE2L2 also being positively correlated with M2-type macrophages and other immune checkpoints, such as tumor necrosis factor superfamily member 4 (TNFSF4), programmed cell death protein 1 (PDCD1), cluster of differentiation 244 (CD244), and inducible T cell co-stimulator (ICOS) (Fig. 5). This relationship suggests that NFE2L2 may influence the tumor immune microenvironment through the m^6^A modification process mediated by METTL16.^129^

METTL16 may influence the recruitment, activation, or function of tumor-infiltrating immune cells, thereby shaping the immune landscape within the tumor microenvironment. Large-scale data analysis revealed that the expression of m^6^A regulators, including METTL16, is closely associated with immune cell infiltration in the tumor microenvironment. This includes cancer-associated fibroblasts, myeloid dendritic cells, CD4^+^ T cells, neutrophils, regulatory T cells, CD8^+^ T cells, and macrophages.^130^ In low-grade gliomas, for example, METTL16 expression has been found to be negatively correlated with the presence of CD8^+^ T lymphocytes. This suggests that METTL16 may regulate the tumor microenvironment by influencing specific immune cell populations, potentially affecting tumor immune evasion and modulating anti-tumor immune responses.^129^ Furthermore, in pancreatic ductal adenocarcinoma and gastric adenocarcinoma, METTL16 expression is significantly associated with tumor-infiltrating immune cells such as B cells and CD8^+^ T cells^34^^,^^131^ (Fig. 5). In hepatocellular carcinoma, the HIF-1α/METTL16/lnc-CSMD1-7/RBFOX2 signaling axis has been shown to regulate interactions between tumor cells and the tumor microenvironment, particularly influencing cell behaviors such as epithelial–mesenchymal transition (Fig. 5). These processes, in turn, affect the infiltration and activity of immune cells within the tumor microenvironment.^42^ METTL16-dependent modulation of cytokine transcripts may be influenced by its interaction with MALAT1 (see the subsection “regulation of non-coding RNAs”), thereby indirectly shaping the immune milieu.^132^ METTL16 may regulate the production or secretion of cytokines, chemokines, and other immune mediators, thereby modulating immune cell trafficking, inflammation, and interactions within the tumor microenvironment.

## Clinical implications and therapeutic potential

Inhibiting METTL16 or its downstream pathways presents a promising strategy for cancer therapy, particularly in cancers where METTL16 acts as an oncogene. By specifically targeting METTL16 with small-molecule inhibitors or nucleic acid-based approaches, it is possible to disrupt its oncogenic activity in most tumors where METTL16 plays a key role in promoting tumorigenesis. Moreover, this strategy could enhance the sensitivity of cancer cells to chemotherapy and immunotherapy. Two compounds, VXL and NIL, have been identified as potential METTL16 inhibitors through compound library screening. The candidate molecules are predicted to span both the SAM binding pocket and the adjacent NPPF substrate channel of METTL16. Molecular-dynamics simulations and MM-PBSA free-energy estimations reveal a network of stable hydrophobic contacts. Nonetheless, experimental confirmation of specific target engagement at the biochemical or cellular level is still absent. In addition, NIL is a broad-spectrum kinase inhibitor and therefore carries a high risk of off-target activity.^133^ The activity of another class of inhibitors, aminothiazolones, has been experimentally validated. *In vitro* studies demonstrated that these compounds reduced m^6^A modification levels on the MAT2A-hp1 hairpin structure. In binding assays, METTL16 was incubated with two different RNA probes: MAT2A-hp1, which contains the UACAGAR hairpin motif highly preferred by METTL16, and a GGACU probe, which represents the canonical DRACH motif recognized by the METTL3/14 complex but lacks specific secondary structure. The results showed that the unlabeled GGACU probe was only able to compete off FAM-labeled MAT2A-hp1 at high concentrations. The affinity of aminothiazolones for the GGACU probe was more than 15-fold lower compared with their affinity for MAT2A-hp1, indicating a degree of target selectivity for METTL16 over the METTL3 complex.^134^ This offers a new avenue for exploring the biological functions of METTL16 and assessing its therapeutic potential through small-molecule inhibition. However, these compounds have not yet been tested in xenograft models that are dependent on METTL16. Pharmacokinetics, off-target transcriptome effects, and rescue experiments with METTL16 point mutants remain outstanding. Addressing these gaps will be essential before moving any METTL16 inhibitor beyond proof-of-concept chemistry.

When a target protein contributes to disease progression not only through its catalytic activity but also via non-catalytic mechanisms, and when it possesses a ligandable site or an engineerable tag, proteolysis-targeting chimeras (PROTACs) or molecular glues are particularly advantageous compared with traditional small-molecule inhibitors.^135^ These molecules recruit the target protein to an E3 ubiquitin ligase, resulting in proteasome-mediated degradation and the complete elimination of both its catalytic and non-catalytic functions. In contrast, conventional active-site inhibitors only block enzymatic activity and cannot eliminate scaffold-based signaling or structural roles. Moreover, targeted protein degradation offers several additional advantages, including reversibility, reduced likelihood of resistance mutations, and greater tolerance to point mutations or target overexpression. These features make degraders especially effective for modulating multifunctional or “non-catalytically oncogenic” proteins, offering a more comprehensive and durable therapeutic strategy than inhibition of catalytic pockets alone.^136^, ^137^, ^138^

In addition to small molecules, RNA interference technologies, such as small interfering RNA (siRNA) or short hairpin RNA (shRNA), can be used to knock down METTL16 expression in cancer cells, potentially leading to further suppression of tumor growth and metastasis.^58^^,^^139^^,^^140^ Gene editing tools like CRISPR/Cas9 can be employed to directly regulate the expression or function of METTL16, repair its mutations in tumor cells, and restore normal DNA damage repair capabilities.^58^^,^^64^ Several technical limitations complicate the current understanding of METTL16 biology. Most available data are derived from immortalized cell lines in which METTL16 is either markedly overexpressed or chronically depleted using shRNA or siRNA approaches. Despite their promise, these technologies have notable limitations. METTL16 is functionally intertwined with other m^6^A regulatory factors, including METTL3/14 and FTO; thus, its knockdown can inadvertently alter the expression or activity of these proteins, further complicating the interpretation of downstream effects.^35^^,^^141^ Moreover, when METTL16 levels deviate from their physiological stoichiometry, intracellular protein–protein interactions can become imbalanced, exhausting folding and trafficking machinery and leading to aberrant pathway activation or nonspecific cytotoxicity.^142^^,^^143^ Conversely, in gene knockdown or knockout experiments, the organism can mount a rapid compensatory response by up-regulating homologous or functionally redundant genes. This genetic compensation, often accompanied by epigenetic relaxation triggered by nonsense-mediated mRNA decay, may partially or completely mask the expected loss-of-function phenotype and can even generate biological effects opposite to those observed in knockdown studies.^144^^,^^145^

Acute protein degradation systems, such as degradation tag (dTAG) and auxin-inducible degron (AID) technologies, provide a strategy to overcome these limitations. dTAGV-1 enables direct, rapid, and reversible degradation of target proteins with single-protein specificity. This approach circumvents the time delay associated with siRNA-mediated knockdown, which requires transcriptional and translational depletion. It also avoids common RNAi-related artifacts, including off-target effects, activation of innate immune responses, and compensatory gene regulation, thereby allowing a more precise assessment of downstream functional consequences. Rapid inactivation of endogenous METTL16 through these methods leads to an immediate collapse in transcription and splicing, highlighting the cell's acute dependence on this factor.^146^ Degradation alone is not sufficient to eliminate artefacts; only by combining tunable CRISPR interference, opto- or chemogenetic degradation systems, precise knock-in strategies, and system-level quantitative validation can we reproduce a protein's true physiological activity at the molecular level.^147^ To accurately assess the therapeutic potential and safety profile of METTL16, future studies should establish physiologically relevant platforms such as three-dimensional organoids or immunocompetent *in vivo* models, which can help minimize dosage artifacts and compensatory responses.

**Figure 6:**
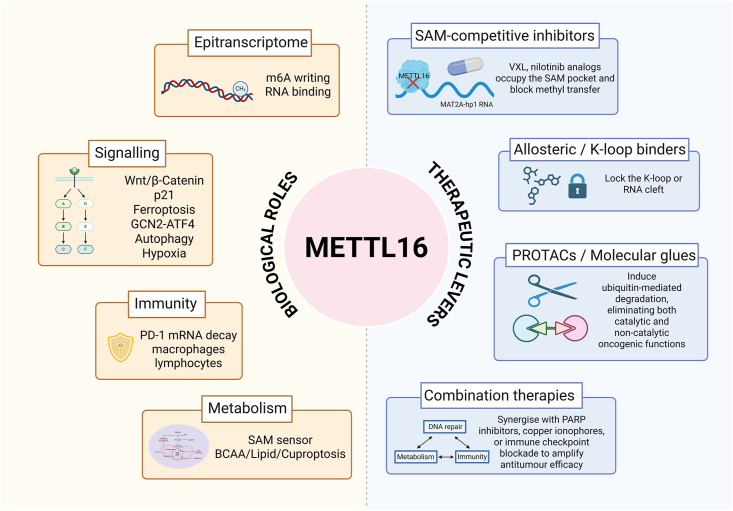
Biological roles of METTL16 and opportunities for therapeutic intervention. The left panel highlights four key tumor-associated functions of METTL16: epitranscriptomic regulation via m^6^A modification and RNA binding; modulation of signaling pathways (*e.g.*, Wnt/β-catenin, p21, ferroptosis, and stress responses); regulation of immune checkpoints and immune cell activity; and metabolic sensing of SAM, along with broader control of amino acid, lipid, and copper homeostasis. The right panel outlines potential therapeutic strategies, including SAM-competitive ligands targeting the catalytic site; allosteric inhibitors or K-loop binders disrupting RNA interaction; PROTACs or molecular glues promoting protein degradation; and combination approaches with PARP inhibitors, copper ionophores, or immune checkpoint blockade.

A graphical overview of METTL16's oncogenic functions and the corresponding therapeutic opportunities is provided in Figure 6. Given METTL16's role in DNA damage repair through RNA methylation, SAM metabolism regulation, and coordination of the cell cycle and DNA repair, a combination of METTL16 inhibitors with PARP inhibitors could effectively target cancer cells with DNA repair deficiencies, thereby enhancing therapeutic efficacy.^56^ METTL16-targeted therapy may complement immunotherapy for cancers characterized by METTL16-driven immunosuppressive microenvironments, but this hypothesis requires further validation.^48^

Notably, studies involving transgenic mouse models with specific knockout of the Mettl16 gene have explored its impact on embryonic development and organ health. These studies have shown that Mettl16 knockout impairs germ cell differentiation in male mice, particularly during the DNA replication phase before meiosis, leading to male infertility. Additionally, the knockout results in a significant reduction in testicular size, disruption of spermatogenesis, and direct impairment of normal testicular tissue function.^148^ Research has also shown that Mettl16 knockout causes developmental arrest during the embryo implantation stage, particularly affecting critical early developmental stages, such as from the 16-cell morula to the 32–64-cell blastocyst stage. This disruption is attributed to the dysregulation of SAM synthetase MAT2A mRNA expression, which subsequently affects the methionine cycle and fundamental metabolic processes essential for cellular activities.^18^ These findings underscore the critical role of Mettl16 in both embryonic development and the health of reproductive organs. The preclinical and clinical validation of METTL16-targeted therapies is still in its infancy, necessitating rigorous studies to fully elucidate their safety, efficacy, and long-term impact. These limitations emphasize the need for continued research to harness METTL16 as a viable and effective target in cancer therapy.

## Conclusion and future directions

Future research into METTL16 holds significant promise, particularly in unraveling the complexities of its RNA binding and methylation activities. While recent advances have identified hundreds of potential m^6^A targets of METTL16, the majority of these remain unvalidated, underscoring the need for comprehensive functional studies. Intriguingly, not all RNAs bound by METTL16 are subject to methylation, suggesting that METTL16's RNA-binding landscape is broader than its methylation scope. This observation highlights METTL16's dual role as both a “writer” and a “reader”. Although only a few direct METTL16 methylation targets have been confirmed, the global reduction in m^6^A levels observed upon METTL16 loss suggests a broader influence on the m^6^A landscape through both direct and indirect mechanisms. Detailed motif analyses have revealed that many of these affected sites are METTL3-dependent, potentially reflecting METTL16's critical role in SAM biosynthesis. Disruption of METTL16 leads to decreased SAM levels, which in turn impacts METTL3-catalyzed methylation events reliant on SAM availability. Future studies should aim to delineate the specific contributions of METTL16 and METTL3 to individual m^6^A sites and to explore whether METTL16 modulates other SAM-dependent methyltransferases targeting RNA, DNA, or proteins. METTL16's functionality is dependent on the higher-order structures of RNA, particularly stem-loop and bulge structures. These structures are abundant in various types of non-coding RNAs, including small RNAs (sRNA), rRNA, transfer RNA (tRNA), and lncRNA. This suggests that METTL16 may broadly participate in the regulation of the expression and functions of non-coding RNAs. The presence of these higher-order structures can influence the binding efficiency and specificity of METTL16, thereby regulating downstream gene expression. Moreover, METTL16 may further contribute to the regulation of biological processes related to cell proliferation, differentiation, and tumorigenesis by affecting the stability and splicing of non-coding RNAs.

The structural biology landscape of METTL16 offers multiple independent entry points for pharmacological intervention (Fig. 6). Its catalytic pocket can be targeted by SAM-competitive ligands, while allosteric binders that stabilize the autoinhibitory K-loop or interfere with the METTL16–RNA interface may provide enhanced selectivity compared with the METTL3/14 complex. In biological contexts where METTL16 promotes tumor progression through non-enzymatic mechanisms, such as eIF3-mediated translational regulation, complete protein degradation using PROTACs or molecular glues may be a more effective strategy. Synthetic lethal approaches also merit systematic investigation. For instance, METTL16 inhibition could be combined with PARP inhibitors in tumors deficient in homologous recombination repair, potentially resulting in tumor-selective lethality. In addition, synergistic therapeutic combinations may further expand the clinical utility of METTL16 targeting. In gastric cancer, for example, inhibition of METTL16 may sensitize tumor cells to copper ionophores, amplifying cuproptosis. Given METTL16's involvement in SAM metabolism, non-coding RNA splicing, and immune checkpoint regulation, pharmacological targeting of this protein has the potential to synergize with existing targeted therapies and immune checkpoint inhibitors, thereby expanding its therapeutic utility. Through the integration of rational drug design and biomarker-informed clinical strategies, METTL16 holds strong potential to transition from an emerging epitranscriptomic regulator to a clinically actionable therapeutic target.

Despite the therapeutic potential of targeting METTL16, several challenges remain, including the development of selective inhibitors with minimal off-target effects, optimization of drug delivery systems to achieve sufficient intracellular concentrations, and overcoming potential resistance mechanisms. Additionally, elucidating the precise molecular mechanisms underlying METTL16-mediated oncogenesis and tumor progression is essential for the rational design of targeted therapies. By addressing these challenges, future research may facilitate the integration of METTL16-targeted therapies into clinical practice and contribute to the advancement of cancer treatment strategies.

## CRediT authorship contribution statement

**Kangjie Qiu:** Writing – original draft, Visualization, Formal analysis, Conceptualization. **Shuxin Zhong:** Conceptualization. **Jinyi Liu:** Conceptualization. **Weini Li:** Visualization. **Cunte Chen:** Writing – original draft. **Yangqiu Li:** Writing – review & editing. **Chengwu Zeng:** Writing – review & editing, Writing – original draft, Supervision, Resources, Conceptualization.

## Funding

This work was supported in part by the 10.13039/501100001809National Natural Science Foundation of China (No. 82370167), 10.13039/501100021171Guangdong Basic and Applied Basic Research Foundation of China (No. 2023A1515012118), and 10.13039/501100010256Guangzhou Municipal Science and Technology Project (China) (No. 2025A03J3231).

## Conflict of interests

The authors declared no competing interests.
